# Impact of Diabetes Mellitus on 30-Day Mortality and Ventilation Outcomes in Critically Ill Patients with Acute Exacerbation of Chronic Obstructive Pulmonary Disease (AECOPD): A Retrospective Cohort Study

**DOI:** 10.3390/life16010036

**Published:** 2025-12-25

**Authors:** Josef Yayan, Kurt Rasche

**Affiliations:** Department of Internal Medicine, Division of Pulmonary, Allergy and Sleep Medicine, HELIOS Clinic Wuppertal, Witten/Herdecke University, Heusnerstraße 40, 42283 Wuppertal, Germany

**Keywords:** acute exacerbation of chronic obstructive pulmonary disease, diabetes mellitus, intensive care unit, 30-day mortality, mechanical ventilation

## Abstract

**Background:** Acute exacerbations of chronic obstructive pulmonary disease (AECOPD) are a major cause of intensive care unit (ICU) admissions and are associated with substantial short-term mortality. Diabetes mellitus is a frequent comorbidity in patients with COPD, yet its impact on short-term outcomes in critically ill AECOPD patients remains uncertain. **Aim:** The aim of this study was to investigate whether diabetes mellitus is independently associated with 30-day mortality in critically ill adult patients admitted to the ICU with AECOPD. **Methods:** We conducted a retrospective cohort study of adult ICU patients with AECOPD using the Medical Information Mart for Intensive Care IV (MIMIC-IV) database. All eligible adult patients with a documented diagnosis of AECOPD during the study period were included. Patients were categorized according to the presence or absence of diabetes mellitus. Diabetes mellitus was identified based on documented diagnostic codes and clinical records at the time of ICU admission. Demographic variables, laboratory parameters obtained within the first 24 h of ICU admission, and mechanical ventilation requirements were assessed. Mechanical ventilation was initiated according to standard clinical indications, including acute respiratory failure, hypoxemia, or hypercapnia. The primary outcome was 30-day all-cause mortality. Kaplan–Meier survival analysis, multivariable logistic regression, and Cox proportional hazards models were applied to identify independent predictors of mortality. **Results:** A total of 5874 ICU patients were included, of whom 2489 (42.3%) had diabetes. Patients with diabetes were slightly younger, more frequently male, and more often received mechanical ventilation than non-diabetic patients. Unadjusted 30-day mortality was lower among diabetic patients (15.3% vs. 17.5%; *p* = 0.032). However, after adjustment for relevant covariates, diabetes was not an independent predictor of 30-day mortality (HR = 0.80; *p* = 0.46). Age, male sex, and elevated lactate levels were associated with increased mortality, while early mechanical ventilation showed an association with improved short-term survival. **Conclusions:** Diabetes mellitus was not independently associated with 30-day mortality in critically ill patients with AECOPD. Short-term outcomes were primarily influenced by age, markers of metabolic stress, and timely ventilatory support. Due to limitations of the database, reliable differentiation between type 1 and type 2 diabetes mellitus and detailed assessment of COPD severity or phenotype were not consistently feasible. Further prospective studies are warranted to clarify the long-term implications of diabetes in this patient population.

## 1. Introduction

Chronic obstructive pulmonary disease (COPD) is a leading cause of morbidity and mortality worldwide and is projected to remain a major contributor to the global disease burden in the coming decades [1]. Acute exacerbations of COPD (AECOPD) are a major driver of hospitalizations and are strongly associated with poor clinical outcomes, including respiratory failure and increased short-term mortality [2,3].

Diabetes mellitus is a common comorbidity in patients with COPD, with reported prevalence rates ranging from 2% to 37%, depending on the population and diagnostic criteria used [4,5]. In critically ill populations, particularly among patients admitted to intensive care units, the prevalence of diabetes is substantially higher due to advanced age, multimorbidity, and metabolic risk profiles. The coexistence of COPD and diabetes mellitus may adversely influence clinical outcomes through several pathophysiological mechanisms, including systemic inflammation, oxidative stress, impaired immune response, hypoxemia, and metabolic dysregulation [6,7]. Moreover, frequent use of systemic and inhaled corticosteroids in COPD management can induce hyperglycemia and exacerbate insulin resistance, further complicating the clinical course in patients with pre-existing diabetes [8].

Several observational studies have reported associations between hyperglycemia, poorly controlled diabetes, and worse outcomes in critically ill patients, including higher rates of hospitalization, prolonged length of stay, and increased mortality [9,10,11]. However, the impact of diabetes on short-term outcomes in ICU patients with AECOPD remains unclear. Previous studies have produced inconsistent findings regarding whether diabetes increases mortality risk or modifies the prognostic relevance of other clinical variables in this setting [12,13]. Importantly, outcomes in AECOPD are strongly influenced by disease severity, bronchial obstruction, COPD phenotype, and the burden of associated comorbidities, which are not consistently captured in large critical care databases.

The aim of the present study was to investigate the association between diabetes mellitus and 30-day mortality in critically ill patients with AECOPD. In addition, we examined laboratory parameters, mechanical ventilation requirements, and survival patterns to determine whether diabetes influences short-term outcomes in this high-risk population. By focusing on short-term ICU outcomes, this study aims to clarify whether diabetes independently affects early mortality in patients with severe AECOPD requiring intensive care.

## 2. Materials and Methods

### 2.1. Study Design and Population

This retrospective cohort study included critically ill adult patients (≥18 years) admitted to the intensive care unit (ICU) with a diagnosis of acute exacerbation of chronic obstructive pulmonary disease (AECOPD). Data were obtained from the Medical Information Mart for Intensive Care IV (MIMIC-IV) database, which contains de-identified clinical information from ICU admissions at the Beth Israel Deaconess Medical Center, Boston, MA, USA, between 2008 and 2019. Only the first ICU admission for each patient during the study period was included to avoid duplication. Patients were categorized into two groups based on the documented presence or absence of diabetes mellitus.

All adult ICU patients with a documented diagnosis of AECOPD during the study period were eligible for inclusion. Exclusion criteria were age <18 years, ICU length of stay <24 h, missing mortality data, or incomplete demographic information.

The relatively high prevalence of diabetes mellitus in this cohort reflects the advanced age and high comorbidity burden typical of critically ill ICU populations rather than the general COPD population.

Due to limitations of the database, spirometric measures of COPD severity (e.g., GOLD stage), bronchial obstruction grading, and detailed COPD phenotypes such as emphysema or bronchiectasis were not consistently available and therefore could not be analyzed.

### 2.2. Data Collection

Demographic characteristics (age and sex), clinical data (diabetes status and requirement for mechanical ventilation), and laboratory measurements obtained within the first 24 h of ICU admission were extracted. Laboratory variables included blood glucose, C-reactive protein (CRP), lactate, and white blood cell (WBC) count.

Diabetes mellitus was identified based on documented diagnostic codes and clinical records at the time of ICU admission. Reliable differentiation between type 1 and type 2 diabetes mellitus was not consistently feasible due to heterogeneous coding practices and was therefore not performed.

Because some laboratory measurements demonstrated substantial variability, including extreme outliers typical of heterogeneous ICU testing conditions, these values were examined descriptively and incorporated into multivariable analyses with appropriate caution.

The initiation of mechanical ventilation (non-invasive or invasive) was determined by the treating clinicians based on standard clinical indications, including acute respiratory failure, hypoxemia, hypercapnia, increased work of breathing, or altered mental status.

The primary outcome was 30-day all-cause mortality following ICU admission.

### 2.3. Statistical Analysis

Continuous variables are summarized as mean ± standard deviation (SD) or median with interquartile range (IQR), depending on distribution, and compared using Student’s *t*-test or the Mann–Whitney U test. Categorical variables are presented as frequencies and percentages and compared using the chi-squared test or Fisher’s exact test, as appropriate.

Survival over 30 days was evaluated using Kaplan–Meier curves and compared via the log-rank test. Multivariable logistic regression and Cox proportional hazards models were used to identify independent predictors of 30-day mortality. Variables with a *p*-value < 0.10 in univariate analyses or considered clinically relevant were included in multivariable modeling. Hazard ratios (HRs) and 95% confidence intervals (CIs) were calculated. All statistical tests were two-sided, and a *p*-value < 0.05 was considered statistically significant. Analyses were performed using VassarStats (Vassar College, Poughkeepsie, NY, USA; accessed 15 September 2025), an established online statistical computation platform.

## 3. Results

A total of 5874 ICU patients with acute exacerbation of COPD (AECOPD) were included in the analysis, comprising 2489 patients with diabetes mellitus and 3385 patients without diabetes. Baseline characteristics are shown in Table 1. Patients with diabetes were slightly younger (67.5 ± 11.3 vs. 69.1 ± 11.9 years; *p* < 0.001), more frequently male (54.6% vs. 52.1%; *p* < 0.001), and more often required mechanical ventilation (47.9% vs. 41.6%; *p* < 0.001) compared with non-diabetic patients. Unadjusted 30-day mortality was lower in the diabetic group (15.3% vs. 17.5%; *p* = 0.032).

The higher rate of mechanical ventilation among patients with diabetes suggests earlier recognition of respiratory deterioration and more frequent escalation of respiratory support in this subgroup.

Laboratory findings stratified by diabetes status are summarized in Table 2. No statistically significant differences were observed in CRP, lactate, or white blood cell (WBC) counts between the two groups. Glucose and lactate measurements showed substantial variability, including extreme outliers commonly observed in heterogeneous ICU populations; therefore, these variables were interpreted with caution and were not used for direct group comparison.

When stratified by 30-day mortality, patients who died had higher glucose and lactate levels compared with survivors, whereas CRP and WBC counts did not differ significantly (Table 3). These findings suggest an association between metabolic stress markers and short-term mortality in critically ill patients with AECOPD.

Multivariable logistic regression analysis identified age (β = 0.071; *p* = 0.020) and lactate (β = 0.238; *p* = 0.025) as independent predictors of 30-day mortality. Diabetes status, glucose, CRP, WBC, sex, and mechanical ventilation were not independently associated with mortality in this model (Table 4). These findings indicate that metabolic stress and increasing age, rather than diabetes status, were the primary determinants of short-term mortality in this cohort.

The Cox proportional hazards model confirmed that age (HR = 1.02 per year; *p* < 0.005), male sex (HR = 3.39; *p* < 0.005), and lactate levels (HR = 1.11; *p* < 0.005) were significantly associated with increased mortality, while WBC count was inversely associated with mortality (HR = 0.95; *p* < 0.005). Mechanical ventilation was associated with improved survival (HR = 0.57; *p* = 0.01). Consistent with the logistic regression findings, diabetes mellitus was not significantly associated with 30-day mortality (HR = 0.80; *p* = 0.46; Table 5). These results further support that short-term mortality was primarily driven by age, sex, and markers of metabolic stress rather than by diabetes status.

**Figure 1:**
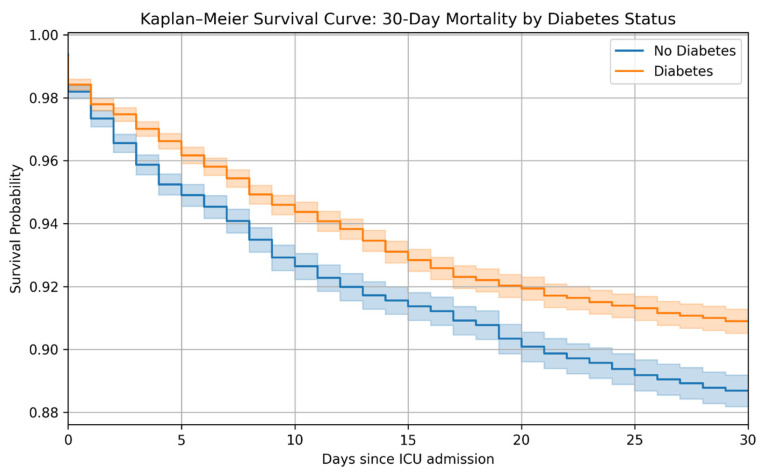
Kaplan–Meier survival curve comparing 30-day mortality between patients with and without diabetes mellitus. Kaplan–Meier survival estimates over a 30-day period comparing intensive care unit (ICU) patients with acute exacerbation of chronic obstructive pulmonary disease (AECOPD) stratified by diabetes mellitus status. The orange curve represents patients with diabetes, and the blue curve represents patients without diabetes. The log-rank test was used to compare survival distributions.

Kaplan–Meier survival curves demonstrated significantly higher 30-day survival among patients with diabetes compared with non-diabetic patients (Figure 1; log-rank *p* < 0.001). This difference reflects unadjusted survival and should be interpreted in the context of multivariable analyses.

**Figure 2:**
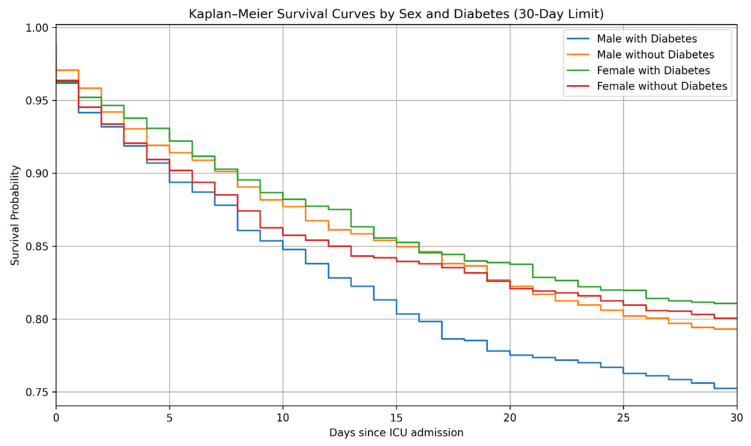
Kaplan–Meier survival curves for 30-day mortality stratified by sex and diabetes status. Kaplan–Meier survival estimates over a 30-day period stratified by sex and diabetes mellitus status in intensive care unit (ICU) patients with acute exacerbation of chronic obstructive pulmonary disease (AECOPD). Survival distributions were compared using the log-rank test (*p* < 0.001).

When stratified by sex, male patients with diabetes exhibited the lowest survival probability, while female diabetic patients showed the highest survival (Figure 2; log-rank *p* < 0.001).

**Figure 3:**
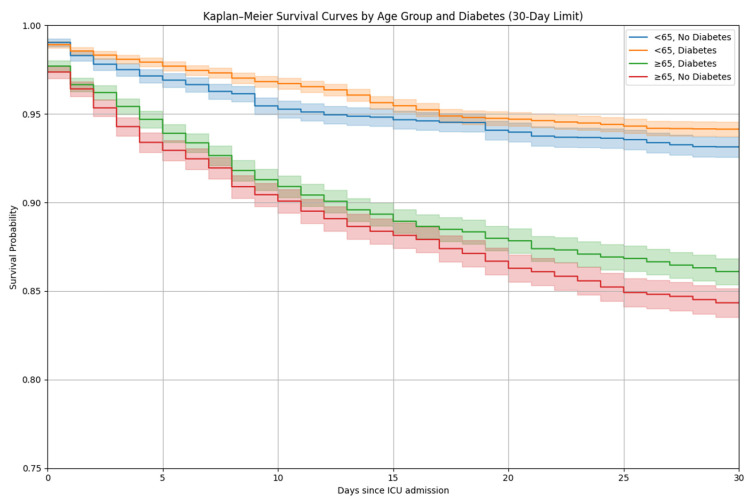
Kaplan–Meier survival curves for 30-day mortality stratified by age group and diabetes status. Kaplan–Meier survival estimates over a 30-day period stratified by age group (<65 years and ≥65 years) and diabetes mellitus status in intensive care unit (ICU) patients with acute exacerbation of chronic obstructive pulmonary disease (AECOPD). Survival distributions were compared using the log-rank test.

Age-stratified analyses indicated that patients aged ≥65 years with diabetes had the poorest survival, whereas patients <65 years without diabetes had the most favorable outcomes (Figure 3). These results represent unadjusted survival patterns across age and diabetes subgroups.

## 4. Discussion

In this retrospective cohort study of critically ill patients with acute exacerbation of chronic obstructive pulmonary disease (AECOPD), we found that diabetes mellitus was not independently associated with 30-day mortality after ICU admission. Although patients with diabetes were slightly younger, more frequently male, and more often required mechanical ventilation than non-diabetic patients, their short-term survival was comparable—and even slightly better—in unadjusted analyses. This observation is consistent with previous reports suggesting a neutral or paradoxically protective effect of diabetes in certain forms of acute respiratory failure [14,15].

The apparent survival advantage observed in diabetic patients in the univariate Kaplan–Meier analysis may reflect several factors, including selection bias and differences in early clinical management. Diabetic patients in our cohort more frequently received ventilatory support, potentially indicating earlier recognition of respiratory compromise and more timely intervention [16]. Importantly, these survival differences were no longer evident after multivariable adjustment, underscoring that the unadjusted Kaplan–Meier findings should be interpreted cautiously. Moreover, some studies have proposed that chronic hyperglycemia may induce metabolic adaptations that partially attenuate the stress response during critical illness, although this hypothesis remains controversial [17]. After adjustment for age, lactate levels, and other prognostic variables, diabetes was no longer associated with mortality, which aligns with recent large ICU studies showing no independent short-term effect of diabetes on survival [18].

Among the predictors evaluated, age, male sex, and serum lactate were strongly associated with 30-day mortality. Elevated lactate is a well-established marker of tissue hypoperfusion and metabolic stress and is closely linked to illness severity in acute pulmonary and systemic disease [19]. The inverse association between white blood cell (WBC) count and mortality observed in the Cox model may suggest that a preserved inflammatory response is associated with improved short-term outcomes, although this relationship requires further investigation [20].

Mechanical ventilation was associated with reduced 30-day mortality in our analysis. This finding should be interpreted cautiously, as it likely reflects the timely initiation of non-invasive or invasive ventilatory support rather than a direct protective effect of ventilation. Early use of non-invasive ventilation in AECOPD is known to decrease the risk of intubation and improve survival, and our results are consistent with this evidence [21,22]. Thus, mechanical ventilation in this context may serve as a marker of early ICU admission and appropriate escalation of care rather than a therapeutic intervention conferring independent survival benefit.

The lack of an independent effect of diabetes on short-term mortality contrasts with epidemiological data linking diabetes to worse long-term outcomes in COPD [23,24]. It is possible that the adverse metabolic and cardiovascular consequences of diabetes manifest more prominently after hospital discharge, whereas short-term ICU outcomes are primarily driven by the severity of the acute episode, underlying comorbidities, and age [25,26]. Additionally, reliable differentiation between type 1 and type 2 diabetes mellitus was not feasible in this database, which may have obscured subtype-specific associations.

This study has several limitations. First, the retrospective single-center design introduces susceptibility to selection bias and unmeasured confounding. Second, laboratory measurements exhibited substantial variability, including extreme outliers typical of heterogeneous ICU testing conditions. These values were interpreted with caution but may still have influenced model estimates. Third, comprehensive comorbidity indices and detailed COPD severity measures, including spirometric classification or COPD phenotype (e.g., emphysema or bronchiectasis), were not consistently available, limiting the ability to fully adjust for baseline disease severity. Furthermore, differentiation between type 1 and type 2 diabetes mellitus could not be performed reliably due to heterogeneous diagnostic coding. Finally, we focused on 30-day mortality, and long-term outcomes such as readmission, functional recovery, or post-discharge mortality were not assessed.

Despite these limitations, our findings contribute to the ongoing discussion regarding the prognostic relevance of diabetes in critically ill patients with AECOPD. The results suggest that short-term survival is determined predominantly by age, metabolic stress markers, and timely ventilatory support rather than by the presence of diabetes alone. Prospective studies incorporating detailed assessments of glycemic control, diabetes subtype, COPD severity, comorbidity burden, and long-term outcomes are needed to clarify the role of diabetes in this population.

## 5. Conclusions

In this retrospective cohort study of critically ill patients with acute exacerbation of chronic obstructive pulmonary disease (AECOPD), diabetes mellitus was not an independent predictor of 30-day mortality after ICU admission. Short-term survival was primarily influenced by age, serum lactate levels, sex, and timely initiation of mechanical ventilation rather than by diabetes status. Although unadjusted analyses suggested a modest survival advantage among patients with diabetes, this association was no longer evident after multivariable adjustment, indicating that underlying differences in patient characteristics and illness severity likely accounted for the observed variation. These findings highlight that short-term outcomes in severe AECOPD are driven predominantly by acute physiological stress and timely critical care interventions rather than by chronic metabolic comorbidity alone. These findings underscore the importance of early recognition of respiratory failure, careful metabolic monitoring, and appropriate ventilatory support as key determinants of short-term outcomes in AECOPD. Future prospective studies incorporating detailed assessments of diabetes subtype, glycemic control, COPD severity, and long-term clinical trajectories are warranted to better define the influence of diabetes on survival and post-ICU recovery in this patient population.

## Figures and Tables

**Table 1:** Baseline characteristics of patients with and without diabetes mellitus. Baseline demographic characteristics, use of mechanical ventilation, and 30-day mortality of intensive care unit (ICU) patients with acute exacerbation of chronic obstructive pulmonary disease (AECOPD), stratified by diabetes mellitus status.

Variable	Diabetes (*n* = 2489)	No Diabetes (*n* = 3385)	*p*-Value
Age (years) ± SD	67.5 ± 11.3	69.1 ± 11.9	**<0.001**
Male gender	1358 (54.6%)	1764 (52.1%)	**<0.001**
Ventilated	1191 (47.9%)	1409 (41.6%)	**<0.001**
30-day mortality	382 (15.3%)	592 (17.5%)	**0.032**

Abbreviations: SD = standard deviation; *n* = number of patients. Note: all bolded *p*-values indicate statistical significance (*p* < 0.05).

**Table 2:** Laboratory parameters of intensive care unit (ICU) patients with acute exacerbation of chronic obstructive pulmonary disease (AECOPD), stratified by diabetes status. Laboratory parameters (glucose, C-reactive protein (CRP), lactate, and white blood cell count (WBC)) measured within the first 24 h of intensive care unit (ICU) admission in patients with and without diabetes mellitus.

Laboratory Parameter	Diabetes (*n* = 2489)	No Diabetes (*n* = 3385)	*p*-Value
Glucose (mg/dL) ± SD	963.6 ± 25,571.6	230.2 ± 217.2	0.153
CRP (mg/L) ± SD	92.5 ± 89.6	88.0 ± 88.8	0.246
Lactate (mmol/L) ± SD	541.4 ± 26,185.0	3.5 ± 3.0	0.317
WBC (×10^3^/µL) ± SD	10.5 ± 6.4	11.7 ± 10.9	0.376

Abbreviations: CRP = C-reactive protein; SD = standard deviation; WBC = white blood cell count.

**Table 3:** Laboratory parameters by 30-Day mortality status in intensive care unit (ICU) patients with acute exacerbation of chronic obstructive pulmonary disease (AECOPD). Laboratory parameters (glucose, C-reactive protein (CRP), lactate, and white blood cell count (WBC)) measured within the first 24 h of intensive care unit (ICU) admission, stratified by 30-day survival status.

Laboratory Parameter	Survived (*n* = 3970)	Died (*n* = 882)	*p*-Value
Glucose (mg/dL) ± SD	246.4 ± 126.7	230.2 ± 217.2	**<0.001**
CRP (mg/L) ± SD	89.7 ± 87.7	88.0 ± 88.8	0.630
Lactate (mmol/L) ± SD	3.2 ± 2.5	3.5 ± 3.0	**<0.001**
WBC (×10^3^/µL) ± SD	11.8 ± 10.0	11.7 ± 10.9	0.981

Abbreviations: CRP = C-reactive protein; SD = standard deviation; WBC = white blood cell count. Note: significant *p*-values are shown in bold.

**Table 4:** Multivariable logistic regression for 30-Day mortality in intensive care unit (ICU) patients with acute exacerbation of chronic obstructive pulmonary disease (AECOPD). Results of a multivariable logistic regression model evaluating associations between selected clinical variables and 30-day mortality in critically ill patients with acute exacerbation of chronic obstructive pulmonary disease (AECOPD). Regression coefficients (β), 95% confidence intervals, and *p*-values are reported.

Variable	β Coefficient	*p*-Value	95% CI Lower	95% CI Upper
Const	−8.113	**0.002**	−13.246	−2.979
Age	0.071	**0.020**	0.011	0.13
Male	1.67	0.051	−0.009	3.349
Diabetes	−0.715	0.363	−2.253	0.824
Glucose	0.001	0.802	−0.005	0.006
CRP	−0.001	0.738	−0.009	0.006
Lactate	0.238	**0.025**	0.029	0.447
WBC	−0.003	0.932	−0.067	0.061
Ventilated	−0.06	0.925	−1.309	1.189

Abbreviations: CRP = C-reactive protein; CI = confidence interval; WBC = white blood cell count. Note: Significant *p*-values are shown in bold.

**Table 5:** Multivariable Cox proportional hazards model for 30-day mortality in intensive care unit (ICU) patients. Results of a multivariable Cox proportional hazards regression analysis evaluating associations between selected clinical variables and 30-day mortality in critically ill patients. Regression coefficients (coefficient (coef)), hazard ratios (exponentiated coefficient (exp(coef))), standard errors (se(coef)), and *p*-values are reported.

Variable	Coef	exp(coef)	se(coef)	*p*-Value
Diabetes	−0.23	0.80	0.31	0.46
Age	0.02	1.02	0.01	**<0.005**
Male	1.22	3.39	0.26	**<0.005**
Ventilated	−0.56	0.57	0.20	**0.01**
Glucose	−0.0	1.0	0.0	**0.04**
CRP	0.0	1.0	0.0	**0.07**
Lactate	0.1	1.11	0.03	**<0.005**
WBC	−0.05	0.95	0.02	**<0.005**

Abbreviations: CRP = C-reactive protein; WBC = white blood cell count. Note: significant *p*-values are shown in bold.

## Data Availability

The datasets analyzed during the current study are publicly available in the MIMIC-IV database (https://physionet.org/content/mimiciv/, accessed 15 September 2025).
